# Association of Noncontrast Computed Tomography and Perfusion Modalities With Outcomes in Patients Undergoing Late-Window Stroke Thrombectomy

**DOI:** 10.1001/jamanetworkopen.2022.41291

**Published:** 2022-11-11

**Authors:** Guilherme B. F. Porto, Ching-Jen Chen, Sami Al Kasab, Muhammed Amir Essibayi, Eyad Almallouhi, Zachary Hubbard, Reda Chalhoub, Ali Alawieh, Ilko Maier, Marios-Nikos Psychogios, Stacey Q. Wolfe, Pascal Jabbour, Ansaar Rai, Robert M. Starke, Amir Shaban, Adam Arthur, Joon-Tae Kim, Shinichi Yoshimura, Jonathan Grossberg, Peter Kan, Isabel Fragata, Adam Polifka, Joshua Osbun, Justin Mascitelli, Michael R. Levitt, Richard Williamson, Daniele G. Romano, Roberto Crosa, Benjamin Gory, Maxim Mokin, Kaustubh S. Limaye, Walter Casagrande, Mark Moss, Ramesh Grandhi, Albert Yoo, Alejandro M. Spiotta, Min S. Park

**Affiliations:** 1 Department of Neurosurgery, Medical University of South Carolina, Charleston; 2 Department of Neurosurgery, Thomas Jefferson University, Philadelphia, Pennsylvania; 3 Department of Neurosurgery, Emory University, Atlanta, Georgia; 4 Department of Neurology, University Medical Center Göttingen, Göttingen, Germany; 5 Department of Radiology, University of Basel, Basel, Switzerland; 6 Department of Neurosurgery, Wake Forest University, Winston-Salem, North Carolina; 7 Department of Radiology, West Virginia University, Morgantown; 8 Department of Neurosurgery, University of Miami Health System, Miami, Florida; 9 Department of Neurology, University of Iowa, Iowa City; 10 Department of Neurosurgery, Semmes-Murphey Neurologic and Spine Clinic, University of Tennessee Health Science Center, Memphis; 11 Department of Neurology, Chonnam National University Hospital, Gwangju, Korea; 12 Department of Neurosurgery, Hyogo College of Medicine, Nishinomiya, Hyogo, Japan; 13 Department of Neurosurgery, University of Texas Medical Branch, Galveston; 14 Neuroradiology Department, Hospital São José Centro Hospitalar, Lisboa, Portugal; 15 Department of Neurosurgery, University of Florida, Gainesville; 16 Department of Neurological Surgery, Washington University in St Louis, St Louis, Missouri; 17 Department of Neurosurgery, University of Texas Health Science Center at San Antonio, San Antonio; 18 Department of Neurosurgery, University of Washington, Seattle; 19 Department of Neurosurgery, Allegheny Health Network, Pittsburgh, Pennsylvania; 20 Department of Radiology, A.O.U.S. Giovanni di Dio e Ruggi d’Aragona, Salerno, Italy; 21 Department of Neurosurgery, Endovascular Neurological Center, Montevideo, Uruguay; 22 Department of Diagnostic and Therapeutic Neuroradiology, Centre Hospitalier Régional Universitaire de Nancy, Nancy, France; 23 Department of Neurosurgery, University of South Florida, Tampa; 24 Department of Neurology, Indiana University, Indianapolis; 25 Department of Cerebrovascular and Endovascular Neurosurgery, Hospital Juan Fernandez, Buenos Aires, Argentina; 26 Department of Interventional Neuroradiology, Washington Regional Medical Center, Fayetteville, Arkansas; 27 Department of Neurosurgery, University of Utah, Salt Lake City; 28 Department of Neurosurgery, Texas Stroke Institute, Plano; 29 Department of Neurosurgery, University of Virginia Health, Charlottesville

## Abstract

**Importance:**

There is substantial controversy with regards to the adequacy and use of noncontrast head computed tomography (NCCT) for late-window acute ischemic stroke in selecting candidates for mechanical thrombectomy.

**Objective:**

To assess clinical outcomes of patients with acute ischemic stroke presenting in the late window who underwent mechanical thrombectomy stratified by NCCT admission in comparison with selection by CT perfusion (CTP) and diffusion-weighted imaging (DWI).

**Design, Setting, and Participants:**

In this multicenter retrospective cohort study, prospectively maintained Stroke Thrombectomy and Aneurysm (STAR) database was used by selecting patients within the late window of acute ischemic stroke and emergent large vessel occlusion from 2013 to 2021. Patients were selected by NCCT, CTP, and DWI. Admission Alberta Stroke Program Early CT Score (ASPECTS) as well as confounding variables were adjusted. Follow-up duration was 90 days. Data were analyzed from November 2021 to March 2022.

**Exposures:**

Selection by NCCT, CTP, or DWI.

**Main Outcomes and Measures:**

Primary outcome was functional independence (modified Rankin scale 0-2) at 90 days.

**Results:**

Among 3356 patients, 733 underwent late-window mechanical thrombectomy. The median (IQR) age was 69 (58-80) years, 392 (53.5%) were female, and 449 (65.1%) were White. A total of 419 were selected with NCCT, 280 with CTP, and 34 with DWI. Mean (IQR) admission ASPECTS were comparable among groups (NCCT, 8 [7-9]; CTP, 8 [7-9]; DWI 8, [7-9]; *P* = .37). There was no difference in the 90-day rate of functional independence (aOR, 1.00; 95% CI, 0.59-1.71; *P* = .99) after adjusting for confounders. Symptomatic intracerebral hemorrhage (NCCT, 34 [8.6%]; CTP, 37 [13.5%]; DWI, 3 [9.1%]; *P* = .12) and mortality (NCCT, 78 [27.4%]; CTP, 38 [21.1%]; DWI, 7 [29.2%]; *P* = .29) were similar among groups.

**Conclusions and Relevance:**

In this cohort study, comparable outcomes were observed in patients in the late window irrespective of neuroimaging selection criteria. Admission NCCT scan may triage emergent large vessel occlusion in the late window.

## Key Points

**Question:**

Do patients undergoing late-window stroke thrombectomy achieve similar 90-day outcomes irrespective of image modality selection?

**Findings:**

This cohort study enrolled 733 patients in the late window undergoing thrombectomy: 419 selected with noncontrast computed tomography (CT); 280, CT perfusion; and 34, diffusion-weighted imaging. After adjustments for confounders, there was no difference in functional independence rates between groups.

**Meaning:**

These findings suggest late-window stroke thrombectomy patient selection via noncontrast CT is associated with comparable outcomes with perfusion modalities.

## Teaser

This cohort study evaluates the association of noncontrast computed tomography and perfusion modalities with outcomes in patients undergoing late-window stroke thrombectomy.

## Introduction

Rapid restoration of blood flow to intracranial circulation is critical for good outcomes in patients with acute ischemic stroke (AIS). Multiple randomized clinical trials have demonstrated the role of mechanical thrombectomy (MT) in patients with anterior circulation emergent large vessel occlusion (ELVO).^1,2^ Initially approved for patients within a 6-hour window, the MT therapeutic window has been extended to 24 hours, with the use of computed tomography perfusion (CTP) and diffusion-weighted imaging (DWI) to select patients for MT according to the results of DAWN (DWI or CTP Assessment with Clinical Mismatch in the Triage of Wake-Up and Late Presenting Strokes Undergoing Neurointervention with Trevo) and DEFUSE 3 (Diffusion and Perfusion Imaging Evaluation for Understanding Stroke Evolution) trials.^3,4,5,6^

The assessment of penumbral tissue as well as core infarct has been reported to correlate with clinical outcomes and therefore assist in selecting appropriate candidates for MT, establishing the amount of irreversible infarct and potential salvageable tissue.^7,8,9^ Patients ideally present with a small core infarct and a much larger penumbral area; in such cases, timely reperfusion will halt progression of the infarct and lead to functional recovery of the remaining salvageable tissue. According to the selection criteria used for the early and late MT trials, patients in early window presentation (0-6 hours) are evaluated for MT candidacy according to a noncontrast computed tomography (NCCT) scan, yet late-window presentation (6-24 hours) candidacy is usually assessed by advanced imaging with perfusion studies.^4,5,6,10^ Although the use of perfusion imaging helps better identify patients who are likely to benefit, there is growing evidence showing that relying on perfusion imaging likely leads to overselection and potentially leads to excluding patients who could benefit from MT.^11^ Furthermore, advanced imaging is not readily available in all stroke centers. In the late window, although infarct core and penumbra can be assessed with a variety of imaging modalities, early ischemic changes can be assessed on NCCT with moderate interrater reliability and estimation of good outcome using Alberta Stroke Program Early CT Score (ASPECTS).^12^ The CLEAR study by Nguyen et al^13^ demonstrated potential widespread use of NCCT in triaging ELVO in the late window. Despite these recent data, there is substantial controversy with regards to the adequacy and use of NCCT for late-window AIS in selecting for candidates for MT.^14^

The objective of this study was to assess clinical outcomes of patients with AIS presenting in the late window who underwent MT stratified by NCCT on admission in comparison to selection by CTP and DWI. We hypothesized that selection of candidacy for MT via NCCT leads to comparable 90-day outcomes as selection of candidacy for MT via CTP or DWI.

## Methods

Prospectively maintained registries of 28 stroke centers in the Stroke Thrombectomy and Aneurysm (STAR) collaboration were included in this study, approved by each individual institution’s institutional review board. A waiver of informed consent was granted due to the retrospective nature of the study and research involving no more than minimal risk due to completely anonymized nature of the data collection. This report follows the Strengthening the Reporting of Observational Studies in Epidemiology (STROBE) reporting guideline for cohort studies. The study population consisted of consecutive patients with stroke who underwent an NCCT head with CT angiogram with or without CTP or DWI and subsequent MT within the predefined late-window treatment period (beyond 6 hours from last known well) between 2013 and 2021 in comprehensive stroke centers across US, Europe, Asia, and South America. The study exposure was selection by NCCT, CTP, or DWI. CTP was selected if the patient underwent NCCT and CTP, and DWI was selected if patient underwent CTP and DWI. Eligibility criteria consisted of patient selection for the procedure via published criteria for thrombectomy: National Institutes of Health Stroke Scale (NIHSS) score of 6 or higher, prestroke modified Rankin scale (mRS) of 0 to 2, anterior circulation large-vessel (internal carotid artery, middle cerebral artery M1 segment, or middle cerebral artery M2 segment) occlusion, and time from time last known well to arterial puncture of 6 to 24 hours.^4,5,15^ Exclusion criteria included large-vessel occlusion presentation in early window (0-6 hours from last known well), posterior circulation large-vessel occlusion, and prestroke mRS 3 to 5. Patients were followed up for 90 days after thrombectomy. All procedures were performed by experienced interventionalists with commercially available aspiration catheters and stent retrievers.

Patient demographic characteristics; comorbidities; large vessel occlusion site; ASPECTS on arrival; admission, discharge, and 90-day NIHSS and mRS; modified thrombolysis in cerebral infarction recanalization score (successful recanalization 2b/3); symptomatic intracerebral hemorrhage (sICH); and intravenous thrombolytics use variables were collected for analyses. sICH was defined using European Cooperative Acute Stroke Study III.^16^ These variables were collected via electronic medical record. Race and ethnicity information (Black, Hispanic, White, other) was collected via electronic medical record. Race and ethnicity were assessed due to known health disparities in stroke care and outcomes. Imaging was reviewed by each enrolling site. Bias was reduced by the multicenter source of the data. Study size was obtained by analyzing all-comers in study enrollment period meeting inclusion criteria.

Primary outcome of the study was functional independence at 90 days (mRS 0-2). The secondary outcomes included shift analysis toward better outcomes, development of sICH, and 90-day mortality.

### Statistical Analysis

All baseline characteristics and outcomes were stratified by imaging modality into 3 groups: NCCT, CTP, and DWI. Continuous variables were reported as median with the IQR. Continuous variables were analyzed using student *t* test and Mann-Whitney U or Wilcoxon rank sum test. Categorical variables were reported as percentage and analyzed using Pearson χ^2^ test or Fisher exact test. We investigated the univariate and multivariable correlations between only functional outcomes as the primary outcome and variables of interest as covariates, which included sex, age, NIHSS on admission, ASPECTS, race, prestroke mRS, hypertension, atrial fibrillation, diabetes, hyperlipidemia, congestive heart failure, previous stroke, smoking history, intravenous thrombolytic, and site of occlusion. Probability of functional independence (mRS 0-2) at 90 days was estimated using binomial logistic regression model. Distribution of 90-day mRS score analyses (ordinal shift) were analyzed using multinomial ordinal logistic regression in which a shift to the value in the lower order was considered a better outcome. logistic regressions, crude and adjusted odds ratios (ORs) and 95% CIs were reported for each parameter. *P* < .05 was considered significant, and all tests were 2-tailed. Regarding patients with missing values, given that the missing data are not at random due to the multicenter nature of this study, no imputation was performed. Missing data were dropped out for each outcome in the analysis. All statistical analyses were performed by JAMOVI open-source R based statistical software version 2.3 (R Project for Statistical Computing). Data were analyzed from November 2021 to March 2022.

## Results

### Baseline Characteristics, Imaging Modalities, Metrics, and Outcomes

A total of 3356 patients in the STAR database were reviewed and 733 (21.8%) met inclusion criteria of undergoing MT due to an emergent large vessel occlusion in the late window (median [IQR] age 69 [58-80] years; 392 [53.5%] female; 449 [65.1%] White). Of those, 581 patients (79.3%) had ASPECTS of 6 or greater. Demographic and clinical characteristics of the included participants were stratified by all-comer candidates for MT in the late window selected by the 3 imaging modalities: NCCT with angiography (419 [57%]), CTP (280 [38%]), DWI (34 [5%]). Demographic data for the cohort are described in Table 1.

**Table 1.** Baseline Characteristics, Metrics, and Outcomes of Patients, According to Imaging Modality Selection for Thrombectomy

Characteristics	Participants, No. (%)				*P* value
	NCCT (n = 419)	CTP (n = 280)	DWI (n = 34)	Total (N = 733)	
Age, median (IQR), y	68 (57-78)	70 (60-81)	75 (70-84)	69 (59-80)	.005^a^
NIHSS on admission, median (IQR)	15 (10-19)	14 (8-19)	15 (10-19)	14 (9-19)	.23^a^
ASPECTS on admission, median (IQR)	8 (7-9)	8 (7-9)	8 (6-10)	8 (7-9)	.37^a^
Sex					
Female	226 (53.9)	146 (52.1)	20 (58.8)	392 (53.5)	.73^b^
Male	193 (46.1)	134 (47.9)	14 (41.2)	341 (46.5)	
Race and ethnicity^c^					
Black	94 (24.8)	29 (10.4)	0	123 (17.8)	<.001^b^
Hispanic	12 (3.2)	22 (7.9)	0	34 (4.9)	
White	237 (62.5)	207 (74.2)	5 (15.6)	449 (65.1)	
Others^d^	36 (9.5)	21 (7.5)	27 (84.4)	84 (12.2)	
Prestroke mRS					
0	319 (76.1)	186 (66.4)	26 (76.5)	531 (72.4)	.048^b^
1	70 (16.7)	67 (23.9)	4 (11.8)	141 (19.2)	
2	30 (7.2)	27 (9.6)	4 (11.8)	61 (8.3)	
Hypertension	280 (66.8)	212 (75.7)	22 (64.7)	514 (70.1)	.03^b^
Atrial fibrillation	141 (33.7)	102 (36.4)	15 (44.1)	258 (35.2)	.40^b^
Diabetes	114 (27.2)	80 (28.6)	8 (23.5)	202 (27.6)	.80^b^
Hyperlipidemia	187 (44.6)	147 (52.5)	14 (41.2)	348 (47.5)	.09^b^
Congestive heart failure	39 (11.9)	24 (8.7)	5 (14.7)	68 (10.6)	.33^b^
Previous stroke	66 (15.8)	49 (17.5)	7 (20.6)	122 (16.6)	.68^b^
Smoking history					
Current	86 (22.2)	59 (22.6)	1 (2.9)	146 (21.4)	<.001^b^
Former	77 (19.8)	74 (28.4)	4 (11.8)	155 (22.7)	
Never	225 (58.0)	128 (49.0)	29 (85.3)	382 (55.9)	
Intravenous thrombolytic	59 (14.1)	40 (14.3)	4 (11.8)	103 (14.1)	.92^b^
Occluded vessel					
Internal carotid artery	140 (33.4)	97 (34.6)	9 (26.5)	246 (33.6)	.06^b^
Middle cerebral artery M1	178 (42.5)	137 (48.9)	20 (58.8)	335 (45.7)	
Middle cerebral artery M2	101 (24.1)	46 (16.4)	5 (14.7)	152 (20.7)	
Onset to puncture time, median (IQR), min	662 (466-931)	742 (490-1020)	566 (481-796)	682 (475-977)	.04^a^
Final thrombolysis in cerebral infarction					
0-2a	66 (15.8)	30 (10.9)	2 (6.2)	98 (13.5)	.08^b^
2b-3	351 (84.2)	246 (89.1)	30 (93.8)	627.0 (86.5)	
Craniectomy	5 (2.7)	2 (0.9)	2 (6.1)	9 (2.0)	.09^b^
NIHSS, median (IQR)					
At 24 h	10 (5-18)	11 (4-17)	13 (8-15)	11 (5-17)	.80^a^
At discharge	6 (2-16)	6 (2-12)	6 (4-10)	6 (2-14)	.38^c^
mRS at discharge, median (IQR)	4 (2-5)	4 (2-5)	4 (2-5)	4 (2-5)	.51^a^
Length of stay, median (IQR), d	7 (4-10)	7 (4-10)	10 (8-15)	7 (4-10)	.22^a^
NIHSS at 90 d, median (IQR)	3 (1-8)	2 (0-5)	2 (1-4)	2 (1-7)	.17^a^
mRS at 90 d, median (IQR)	3 (1-5)	3 (1-5)	4 (2-5)	3 (1-5)	.95^a^
mRS at 90 d					
0-2	92 (37.9)	83 (35.3)	9 (34.6)	184 (36.5)	.83^a^
3-6	151 (62.1)	152 (64.7)	17 (65.4)	320 (63.5)	
Symptomatic intracerebral hemorrhage	34 (8.6)	37 (13.5)	3 (9.1)	74 (10.5)	.12^e^
Mortality at 90 d	78 (27.4)	38 (21.1)	7 (29.2)	123 (25.2)	.29^a^

Abbreviations: ASPECTS, Alberta Stroke Program Early CT Score; CTP, computed tomography perfusion; DWI, diffusion-weighted imaging; mRS, modified Rankin scale; NCCT, noncontrast head computed tomography; NIHSS, National Institutes of Health Stroke Scale.

^a^ Mann-Whitney U or Wilcoxon rank sum test.

^b^ Pearson χ^2^ test.

^c^ Race classifications were self-reported and retrieved from electronic medical record.

^d^ Others included American Indian, Asian, Native Hawaiian or other Pacific Islander.

^e^ Fisher exact test.

The median (IQR) age was different among the 3 categories: (NCCT, 68 [57-78] years; CTP, 70 [60-81] years; DWI, 75 [70-84] years; *P* < .005). There was no difference in median (IQR) ASPECTS on admission (NCCT, 8 [7-9]; CTP, 8 [7-9]; DWI, 8 [7-9]; *P* = .37) or median (IQR) NIHSS on admission (NCCT, 15 [10-19]; CTP, 14 [8-19]; DWI, 15 [10-19]; *P* = .23). Prestroke functional status was similar among the 3 groups. There were no differences in comorbidities (hypertension, atrial fibrillation, diabetes, hyperlipidemia, congestive heart failure, or prior stroke). There were fewer current smokers in the DWI category (NCCT, 86 [22.2%]; CTP, 59 [22.6%]; DWI, 1 [2.9%]; *P* < .001). There was no difference in administration of intravenous thrombolytics or site of large vessel occlusion. Median (IQR) symptom onset to groin puncture time was lower in DWI (NCCT, 662 minutes [466-931]; CTP, 742 minutes [490-1020]; DWI, 566 minutes [481-796]; *P* = .04). There was no significant difference in successful recanalization rates among groups (NCCT, 351 [84.2%]; CTP, 246 [89.1%]; DWI, 30 [93.8%]; *P* = .08). The rates of craniotomy, median NIHSS at 24 hours, NIHSS at discharge, mRS at discharge, length of hospital stay, NIHSS at 90 days, mRS at 90 days, sICH, and mortality were not statistically different among the 3 imaging cohorts. Functional independence at 90 days was noted in 184 patients (NCCT, 92 [37.9%]; CTP, 83 [35.3%]; DWI, 9 [34.6%]) (Figure). Symptomatic intracerebral hemorrhage (NCCT, 34 [8.6%]; CTP, 37 [13.5%]; DWI, 3 [9.1%]; *P* = .12) and mortality (NCCT, 78 [27.4%]; CTP, 38 [21.1%]; DWI, 7 [29.2%]; *P* = .29) were similar. Missing data are available eTable in the Supplement.

**Figure.**
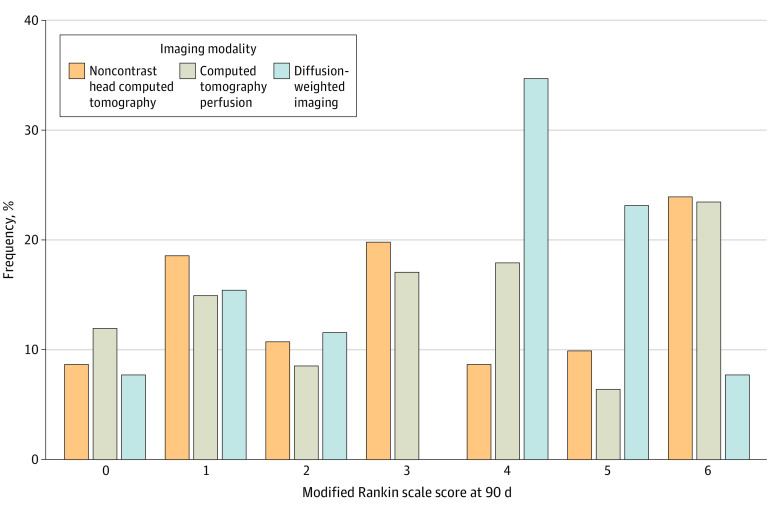
Distribution of 90-Day Modified Rankin Scale Score by Imaging Modality A 0 on the modified Rankin scale indicates no disability; 6, death.

### Analysis of 90-Day Functional Independence

Univariate and multivariable analyses stratified by functional independence (mRS 0-2) at 90 days for all-comers in the late window are shown in Table 2. There was no difference in the 90-day rate of functional independence between NCCT- and CTP-selected patients (aOR, 1.00; 95% CI, 0.59-1.71; *P* = .99), or NCCT- and DWI-selected patients (aOR, 1.33; 95% CI, 0.4-4.35; *P* = .63) after adjusting for confounders. The following variables were independently associated with good outcome at 90 days: age (aOR, 0.97; 95% CI, 0.95-0.99; *P* = .001), NIHSS on admission (aOR, 0.90; 95% CI, 0.86-0.94; *P* < .001), ASPECTS (aOR, 1.20; 95% CI, 1.02-1.42; *P* = .03), prestroke mRS (aOR, 0.13; 95% CI, 0.03-0.63; *P* = .01), hypertension (aOR, 0.51; 95% CI, 0.28-0.93; *P* = .02), diabetes (aOR, 0.57; 95% CI, 0.38-0.87; *P* = .009), and prior stroke (aOR, 0.58; 95% CI, 0.34-0.98; *P* = .04).

**Table 2.** Univariate and Multivariate Predictors of 90-Day Functional Independence

Factor	OR (95% CI)	
	Univariate analysis	Multivariate analysis
Imaging modality		
NCCT	1 [Reference]	1 [Reference]
CTP	0.90 (0.62-1.30)	1.00 (0.59-1.71)
DWI	0.87 (0.37-2.03)	1.33 (0.41-4.35)
Age	0.97 (0.96-0.99)	0.97 (0.95-0.99)
NIHSS on admission	0.88 (0.86-0.91)	0.90 (0.86-0.94)
ASPECTS	1.27 (1.13-1.44)	1.20 (1.02-1.42)
Female sex	1.07 (0.74-1.54)	1.25 (0.75-2.08)
Race and ethnicity		
White	1 [Reference]	1 [Reference]
Black	0.58 (0.33-1.01)	0.46 (0.20-1.06)
Hispanic	1.11 (0.46-2.69)	1.27 (0.41-3.98)
Others	0.89 (0.50-1.56)	0.76 (0.31-1.85)
Prestroke mRS		
0	1 [Reference]	1 [Reference]
1	0.51 (0.32-0.81)	0.64 (0.34-1.21)
2	0.19 (0.72-0.49)	0.13 (0.03-0.63)
Hypertension	0.54 (0.36-0.80)	0.51 (0.28-0.93)
Atrial fibrillation	0.84 (0.57-1.23)	1.33 (0.74-2.40)
Diabetes	0.57 (0.38-0.87)	0.66 (0.36-1.19)
Hyperlipidemia	1.07 (0.74-1.54)	1.43 (0.84-2.43)
Congestive heart failure	0.81 (0.42-1.54)	0.87 (0.34-2.25)
Previous stroke	0.58 (0.34-0.98)	0.73 (0.35-1.49)
Smoking history		
Never	1 [Reference]	1 [Reference]
Current	1.03 (0.63-1.69)	1.01 (0.50-2.01)
Former	1.08 (0.69-1.70)	1.32 (0.70-2.48)
Intravenous thrombolytic	0.74 (0.43-1.30)	0.51 (0.24-1.10)
Site of occlusion		
Internal carotid artery	0.97 (0.65-1.46)	1.05 (0.60-1.85)
Middle cerebral artery M1	1 [Reference]	1 [Reference]
Middle cerebral artery M2	1.27 (0.78-2.06)	0.95 (0.46-1.95)

Abbreviations: ASPECTS, Alberta Stroke Program Early CT Score; CTP, computed tomography perfusion; DWI, diffusion-weighted imaging; mRS, modified Rankin scale; NCCT, noncontrast head computed tomography; NIHSS, National Institutes of Health Stroke Scale; OR, odds ratio.

### Analysis of 90-Day Ordinal mRS Shift Toward Better Outcome

Univariate and multivariable analyses of imaging modality, baseline characteristics, and metrics with 90-day ordinal mRS shift toward better outcomes are shown in Table 3. After adjusting for confounders, there was no difference in 90-day ordinal mRS shift between patients selected by NCCT vs CTP (aOR, 1.06; 95% CI, 0.7-1.61; *P* = .77) and NCCT vs DWI (aOR, 1.86; 95% CI, 0.80-4.31; *P* = .15). ASPECTS (aOR, 1.15; 95% CI, 1.02-1.30; *P* = .02) was associated with a significantly higher odds of a 1-point shift to better outcome. The following variables were associated with significantly lower odds of a 1-point shift to better outcome: age (aOR, 0.96; 95% CI, 0.94-0.97; *P* < .001), NIHSS on admission (aOR, 0.93; 95% CI, 0.90-0.95; *P* < .001), and higher prestroke mRS (aOR, 0.37; 95% CI, 0.18-0.76; *P* = .007).

**Table 3.** Univariate and Multivariate Analysis of Imaging Modality, Baseline Characteristics, and Metrics With 90-Day Ordinal mRS Score Shift Toward Better Outcomes

Factor	OR (95% CI)	
	Univariate analysis	Multivariate analysis
Imaging modality		
NCCT	1 [Reference]	1 [Reference]
CTP	0.98 (0.72-1.35)	1.06 (0.70-1.61)
DWI	0.90 (0.46-1.78)	1.86 (0.80-4.31)
Age	0.96 (0.95-0.97)	0.96 (0.94-0.97)
NIHSS on admission	0.91 (0.88-0.93)	0.93 (0.90-0.95)
ASPECTS	1.22 (1.11-1.33)	1.15 (1.02-1.30)
Female sex	0.97 (0.69-1.40)	1.21 (0.82-1.77)
Race and ethnicity		
White	1 [Reference]	1 [Reference]
Black	0.86 (0.59-1.31)	0.69 (0.39-1.22)
Hispanic	1.79 (0.86-3.78)	1.73 (0.72-4.21)
Others	0.85 (0.54-1.36)	0.55 (0.28-1.09)
Prestroke mRS		
0	1 [Reference]	1 [Reference]
1	0.59 (0.40-0.85)	0.70 (0.44-1.12)
2	0.33 (0.18-0.57)	0.37 (0.18-0.76)
Hypertension	0.68 (0.48-0.95)	0.89 (0.56-1.41)
Atrial fibrillation	0.66 (0.47-0.92)	1.03 (0.66-1.60)
Diabetes	0.66 (0.47-0.92)	0.75 (0.48-1.16)
Hyperlipidemia	1.07 (0.79-1.46)	1.25 (0.85-1.85)
Congestive heart failure	0.88 (0.52-1.50)	1.15 (0.61-2.16)
Previous stroke	0.76 (0.51-1.14)	0.89 (0.53-1.49)
Smoking history		
Never	1 [Reference]	1 [Reference]
Former	1.13 (0.77-1.66)	1.16 (0.72-1.87)
Current	1.14 (0.76-1.72)	0.76 (0.45-1.29)
Intravenous thrombolytic	0.85 (0.54-1.33)	0.62 (0.35-1.07)
Site of occlusion		
Internal carotid artery	0.94 (0.67-1.32)	0.88 (0.58-1.35)
Cervical segment	1 [Reference]	1 [Reference]
Petrous segment	1.14 (0.75-1.74)	1.03 (0.59-1.81)

Abbreviations: ASPECTS, Alberta Stroke Program Early CT Score; CTP, computed tomography perfusion; DWI, diffusion-weighted imaging; mRS, modified Rankin scale; NCCT, noncontrast head computed tomography; NIHSS, National Institutes of Health Stroke Scale; OR, odds ratio.

## Discussion

This cohort study found comparable outcomes among patients with anterior circulation large vessel occlusion presenting in the late window who underwent MT based on NCCT and those who underwent advanced imaging. We found no difference in 90-day functional independence and no difference in 90-day ordinary mRS shift between NCCT and CTP and NCCT and DWI in a large sample of 766 patients who underwent MT in the late window. Our findings provide important data supporting the use of NCCT to triage patients with anterior circulation LVO in the late window. Such a simplified approach to select patients for MT, particularly given the lack of wide availability of perfusion studies, could lead not only to shortened treatment times, but also expand the current guideline indications for MT in the late window in areas where advanced imaging is not available.

These results build on the recently published CLEAR study^13^ showing similar outcomes among patients with intracranial internal carotid artery, middle cerebral artery M1 segment, or middle cerebral artery M2 segment occlusion selected by NCCT compared with perfusion imaging. In the CLEAR study, 534 patients were selected by NCCT, 752 by CTP, and 318 by magnetic resonance imaging (MRI). There were no differences in 90-day rates of functional independence between the NCCT and CTP groups; however, rates were lower in the MRI group in comparison to NCCT.^13^ Both DAWN and DEFUSE 3^3,4,5,6^ trials used perfusion imaging to select patients for MT. The use of advanced imaging was meant to select patients who are most likely to benefit from MT. There has been growing evidence over the past few years showing correlation between NCCT ASPECTS and CTP. A study by Demeestere et al^17^ in a cohort of 59 patients who underwent NCCT, CTP, and MRI within 100 minutes from CT imaging showed no difference in accuracy of CTP and NCCT ASPECTS to estimate hyperacute stroke in MRI. Haussen et al^18^ studied the correlation of NCCT and CTP in a cohort of 332 showing a fair correlation between CTP ischemic core and ASPECTS (*R* = −0.36) and moderate correlation between ASPECTS and final infarct volume (*R* = −0.42) and between CTP ischemic core and final infarct volume (*R* = 0.50). This exemplifies the large variability in the CTP-derived data among different categories of ASPECTS. Besides the lack of wide availability of perfusion imaging, exposure to higher levels of radiation and potential delays in treatment, there are several limitations to perfusion imaging, such as susceptibility for motion artifact, and potential errors in postprocessing algorithms. In the MR. CLEAN trial,^19^ approximately 50% of patients who underwent CTP had reported motion artifact and technical issues rendering the imaging uninterpretable.

NCCT is an essential tool to determine early ischemic changes and infarct volumes, given its simplicity and interrater reliability. It is widely available and has potential to adequately triage patients with emergent LVO in the late window. Computed tomography angiography and CT perfusion continue to be the standard of care for assessing collaterals and infarct volume; however, they can be fraught with concerns, especially due to the known risk of computed tomography angiography underestimating collateral blood flow to penumbra and CTP’s overestimation of final core infarct. Furthermore, Desai et al^20^ studied known methods to evaluate clinical severity of stroke (NIHSS) to calculate the volume of hypoperfused tissue and during an LVO stroke and found that NIHSS can be used as a surrogate to CTP and allow clinical stroke scale and NCCT to become primary determinants of MT eligibility.

### Strengths and Limitations

Our study’s strength is the large number of patients selected across a wide range of hospital systems as well as the prospective nature of the database, with enrollment of consecutive patients presenting to each of the enrolled stroke centers. Our study has limitations: internal carotid artery, middle cerebral artery M1, middle cerebral artery M2, and ELVO only were included, and patients with mRS 0 to 2 before arrival to the hospital were selected. Imaging modality selection is dependent on institutional practice and practitioner decision rather than standardized protocol. The DWI cohort is small and was obtained largely from select centers. Furthermore, each individual center was responsible for interpretation of ASPECTS and CTP and DWI studies, and this can introduce selection bias. In the cohort with NCCT and CTP available or NCCT and DWI available, perfusion imaging was used for clinical decision at the discretion of the neurointerventionalist.

## Conclusions

In a multicenter patient cohort presenting in the late window triaged with NCCT, there was no evidence of clinical outcome differences between NCCT and CTP or DWI triage for ELVO. For this reason, it should be considered part of the assessment for candidacy for MT in patients with AIS presenting beyond the 6-hour window.

## Notes

Supplement. **eTable. **Missing Data for Each Respective Variable at the Time of Data Analysis
