# Supplementary material for: Association of Noncontrast Computed Tomography and Perfusion Modalities With Outcomes in Patients Undergoing Late-Window Stroke Thrombectomy
Source: JAMA Netw Open. 2022 Nov 11;5(11):e2241291. doi: 10.1001/jamanetworkopen.2022.41291 (PMC9652750; doi:10.1001/jamanetworkopen.2022.41291)
Supplement: Supplement. — eTable. Missing Data for Each Respective Variable at the Time of Data Analysis [file jamanetwopen-e2241291-s001.pdf]

Supplemental Online Content

Porto GBF, Chen CJ, Al Kasab S, et al. Association of noncontrast computed tomography and perfusion modalities with outcomes in patients undergoing late-window stroke thrombectomy. *JAMA Netw Open*. 2022;5(11):e2241291. doi:10.1001/jamanetworkopen.2022.41291

eTable. Missing Data for Each Respective Variable at the Time of Data Analysis

This supplemental material has been provided by the authors to give readers additional information about their work.

© 2022 Porto GBF et al. *JAMA Network Open.*

eTable. Missing Data for Each Respective Variable at the Time of Data Analysis

Characteristics	Number of missing data (Tota1 733)
Race ethnicity	43
Congestive heart failure	93
Smoking history	50
Admission NIHSS	1
ASPECTS	88
IV thrombolytics	3
Final TICI	8
Complications	3
Distal embolization	65
Symptomatic ICH	30
Craniectomy	278
NIHSS at 24 hours	125
NIHSS at discharge	168
mRS at discharge	63
Length of stay	263
NIHSS at 90 days	540
mRS at 90 days	229

© 2022 Porto GBF et al. *JAMA Network Open.*
